# Mesenchymal stem cells from tumor microenvironment favour breast cancer stem cell proliferation, cancerogenic and metastatic potential, via ionotropic purinergic signalling

**DOI:** 10.1038/s41598-017-13460-7

**Published:** 2017-10-13

**Authors:** A. Maffey, C. Storini, C. Diceglie, C. Martelli, L. Sironi, C. Calzarossa, N. Tonna, R. Lovchik, E. Delamarche, L. Ottobrini, F. Bianco

**Affiliations:** 1 Neuro-Zone srl, OpenZone Via Ariosto 21, 20091 Bresso (MI), Italy; 2 Sanipedia srl, OpenZone Via Ariosto 21, 20091 Bresso (MI), Italy; 3 Department of Pathophysiology and Transplantation, University of Milan, Via F.lli Cervi 93, 20090 Segrate Milano, Italy; 4 Fondazione Fernando Santarelli, Neuroinflammation Lab, Corso Venezia 18, 20122 Milano, Italy; 5 IBM Research, Zurich, Saeumerstrasse 4, 8803 Rueschlikon Switzerland; 6 Institute for Molecular Bioimaging and Physiology (IBFM), National Research Council (CNR), Via F.lli Cervi 93, 20090 Segrate Milano, Italy; 7 BrainDTech srl, OpenZone Via Ariosto 21, 20091 Bresso (MI), Italy

## Abstract

Interaction between tumor cells and the microenvironment is key in initiation, progression, and invasiveness of cancer. In particular, mesenchymal stem cells (MSCs) are recruited to the sites of developing tumors, thus promoting metastasis formation. Although it is well known that MSCs migrate and integrate in the tumor microenvironment (TME), their fate and function inside the tumor is still not clear. In this study, we analyzed the role played by MSCs in breast cancer oncogenesis. Data indicate that interaction of breast cancer cells with MSCs results in an increased proliferation and metabolic activity of breast cancer cells, partially due to MSC-derived microvesicles that are shed in the TME. Moreover, we addressed the question of whether we could modulate such interaction by acting on P2X-mediated intercellular communication. By inhibiting P2X-mediated purinergic signaling, we succeeded in reducing both the cancerogenic as well as the metastatic potential of breast cancer cells co-cultured with MSCs, in 2D as well as in 3D *in vitro* models. Data obtained demonstrate for the first time that the trophic effect of MSCs on breast cancer cell growth is exerted via ionotropic purinergic signaling, thus suggesting the inhibition of the purinergic signaling system as a potential target for therapeutic intervention.

## Introduction

Breast cancer is recognized as the most prevalent malignancy for women, with significant impact on lifespan and quality of life. Conventional therapies, predominantly surgery, radiation, and chemotherapy, concur in controlling the disease without leading to long-term cure.

The formation of breast carcinomas is often accompanied by a well-orchestrated reaction, which involves the recruitment of a variety of stromal cells with both pro- and anti-tumorigenic activities^1^. Recent findings demonstrate that, among others, cancer formation is a process which involves the recruitment of endogenous mesenchymal stem cells (MSCs), and that such MSCs exert powerful activities within the tumor stroma that affects the biology of the tumor as a whole. Indeed, MSCs within the tumor were shown to enhance, among other things, fibrovascular desmoplasia, tumor formation, and metastasis^2,3^.

One of the most important characteristics of cancer pathogenesis is the metastatic potential, usually leading to a poor prognosis. It has been shown that MSCs favor the invasiveness of cancer cells via deposition of laminin, fibronectin and fibrillar collagen^4^, which increases cancer cell proliferation and invasion^5^. High expression of stromal fibronectin has been associated with negative prognosis in breast cancer^6–9^. MSCs may also play a critical role in extracellular matrix (ECM) remodeling, as the co-culture of MSCs with breast cancer cells causes upregulation of lysyl oxidase^10^, a collagen crosslinker. According to this evidence, it is important to evaluate the metastatic potential in a controlled *in vitro* 3D system, which allows to monitor the formation of mammospheres.

The tumor microenvironment is typically enriched in ATP, deriving from many sources. In particular, MSCs, via microvesicle and exosome release, significantly contribute to the increase in extracellular ATP levels via spontaneous or organelle-mediated release^11^. Recent achievements in measuring extracellular ATP levels, allowed to clearly demonstrate that ATP at site of cancer can reach micromolar concentrations^12,13^.

Recently, the role of purinergic signaling in cancer has been deeply investigated. A link between cancer and purinergic receptor has been demonstrated in many papers and in many cancer types. In particular, the P2X7 receptor is accepted as the main player in cell death, via apoptosis or necrosis, when activated by high (millimolar) levels of ATP. For this reason, potential therapeutic approaches have been focusing on the pharmacological modulation of P2X7. In fact, micromolar levels of ATP at the extracellular site ensure a tonic activation of P2X7, that is linked to an anti-apoptotic and growth-favoring effect^14^. Nevertheless, there’s a growing amount of literature suggesting that the tonic activation of P2X7 receptor is characterized by a trophic, growth-promoting, rather than cytotoxic effect^14,15^. In line with previous studies carried out also by our team, in which the growth promoting role of P2X7 was deeply investigated, in this study we focused our attention on the role of purinergic receptors (and P2X7 in particular) in the development of breast cancer. We took into consideration the pathophysiologic activation of P2 receptors in the tumor microenvironment enriched with human adipose derived MSCs. Adipose derived MSCs are known not to be tumorigenic *per se*, as they are not able to induce a neoplastic transformation of normal mammary cells, however they can exacerbate tumorigenic behaviour, creating an inflammatory microenvironment which sustains tumor growth and angiogenesis^16^.

## Results

### MSCs favour breast cancer cell proliferation, through P2X receptor signalling: a potential role for MVs?

It is well known that the growth rate of cancer is strongly influenced by the interaction of tumor cells with different cell populations in the tumor stroma. In particular, we focused our attention on the modulatory role played by human adult MSCs, which represent a key element in the breast tumor microenvironment (TME).

**Figure 1:**
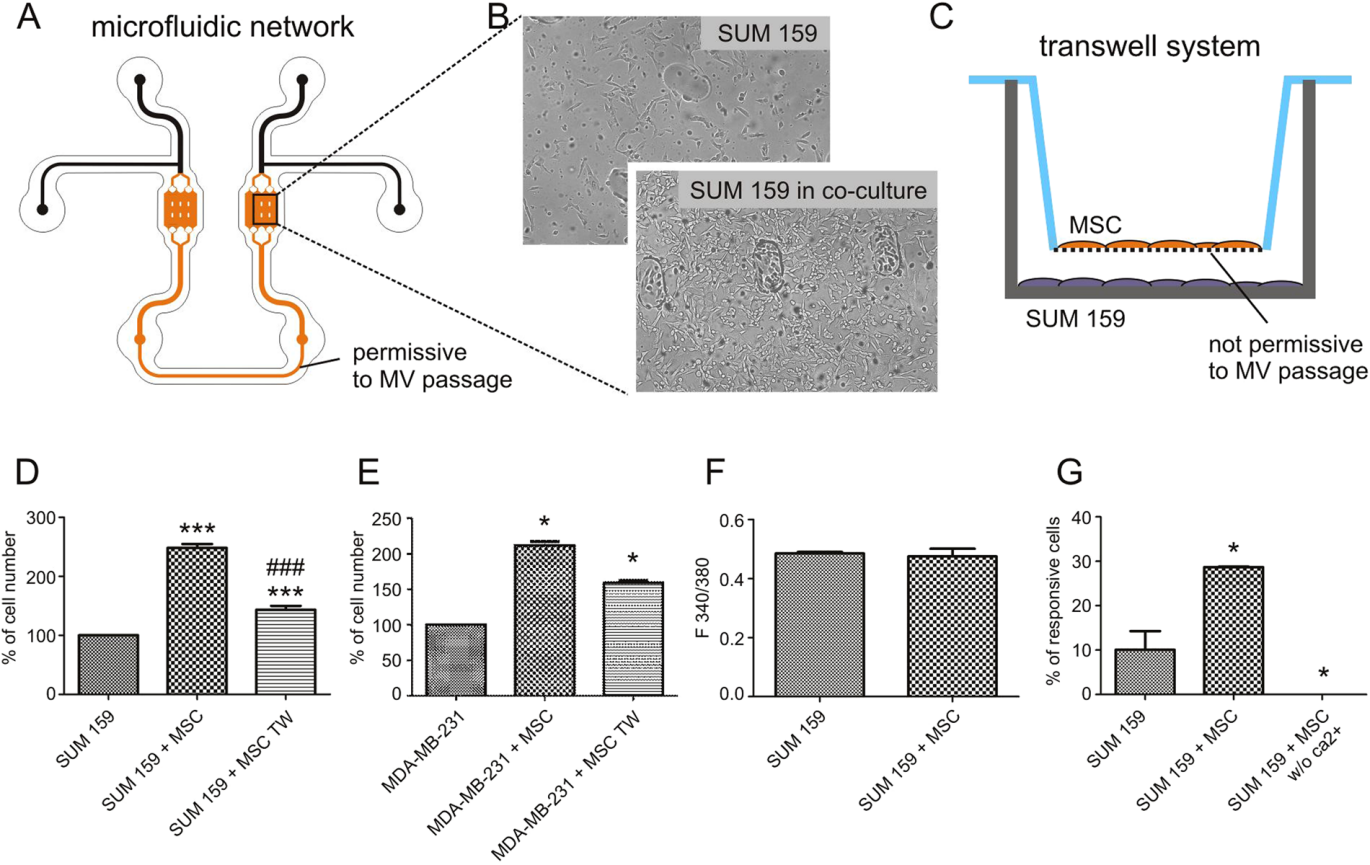
MSC and SUM159PT were cocultured for 48 hours in a culturing system either permissive (**A**) or not permissive (**C**) to microvesicle (MV) passage. When SUM159PT were cocultured in a permissive system, MSC significantly increased cell growth (**B**,**D**). The effect of MSC on cell growth was reduced when a not permissive system was used (SUM159 + MSC TW group, panel D). Similar results were obtained replicating experiments with anouther human breast cancer cell line, MDA-MB-231 (**E**). In order to verify if this effect was mediated by purinergic receptors, calcium dynamics were investigated: no differences in basal intracellular calcium levels of cancer cells was observed with or without MSC (**F**), but the number of cancer cells responsive to exogenous 1 mM ATP significantly increased in the presence of MSC (**G**). One-way ANOVA with Bonferroni as post-hoc test: *p < 0.05 vs SUM159PT; **p < 0.01 vs SUM159PT; ***p < 0.001 vs SUM159PT; ^###^p < 0.001 vs SUM159PT + MSC.

In order to recreate the microenvironmental interaction, we took advantage of a microfluidic setup previously developed in our lab (Fig. 1A), which allows for the fine culturing of different cell types in controlled microenvironments^17^. Human breast cancer cells (SUM159PT) were cultured either independently or in soluble-factor mediated communication with MSCs for 48 hours. SUM159PT co-cultivated in microfluidic communication with MSCs clearly showed an increased proliferation, as demonstrated by the representative images here reported (Fig. 1B).

As MSCs are known to actively modulate intercellular communication via the release of vesicular structures (exosomes and microvesicles) in the surrounding microenvironment^18^, we addressed whether the increased proliferation we observed was mediated, at least partially, from organelles of MSC origin. We have previously shown that the microfluidic set up was permissive to the passage of organelles between chambers^17^ (Fig. 1A) without size limitation; hence, taking advantage of this platform, we recreated the co-culture either using the permissive microfluidic platform, or using transwell systems (0.4μm filter), which were permissive to the passage of exosomes (70–150 nm diameter) but not to microvesicles (MVs) (0.4–1 µm) (Fig. 1C). Quantitative evaluation of data normalized to the number of independently cultured SUM159PT cells, indicated that the observed increase in cancer cell number was induced by MSC conditioned medium, most probably mainly mediated by MSC-derived MVs, given the observed increase was not present when a non-permissive system was used (Fig. 1D). In order to confirm these data, the permissive vs. non-permissive experimental setting was replicated using a different human breast adenocarcinoma cell line MDA-MB-231. Data confirmed an increase in the number of cells in the permissive coculture system with respect to the non-permissive system, although the increase in cell number in the transwell with respect to untreated cells suggest that factors other than shed MVs can also contribute to the observed phenomenon (Fig. 1E).

Malignant tumors such as breast cancer are characterized by a strong inflammatory response, in which ATP accumulates within the TME^12^. It has been demonstrated that the extracellular concentration of ATP is sufficient to keep most P2 receptors in a tonic activation, including P2X7^19^. We therefore investigated the role played by purinergic signaling on cancer cells. While no differences were observed in basal intracellular calcium levels of cancer cells (Fig. 1E), cultured either in soluble mediated co-culture (SUM 159 + MSC) or without MSCs (SUM 159), the number of cancer cells responsive to exogenous 1 mM ATP significantly increased in the presence of MSCs as reported in Fig. 1G, where the percentage of cells responsive to the exogenous ATP stimulations are calculated (Fig. 1G). This increase in SUM159PT ATP responsiveness is most probably due to an increase in the functional response of P2X ionotropic, rather than P2Y metabotropic purinergic receptors, given the response of cancer cells is abolished when cells are challenged with ATP in the absence of extracellular calcium (Fig. 1G).

### P2X7 signalling inhibition reduces MSC-mediated increased proliferation in breast cancer cells

**Figure 2:**
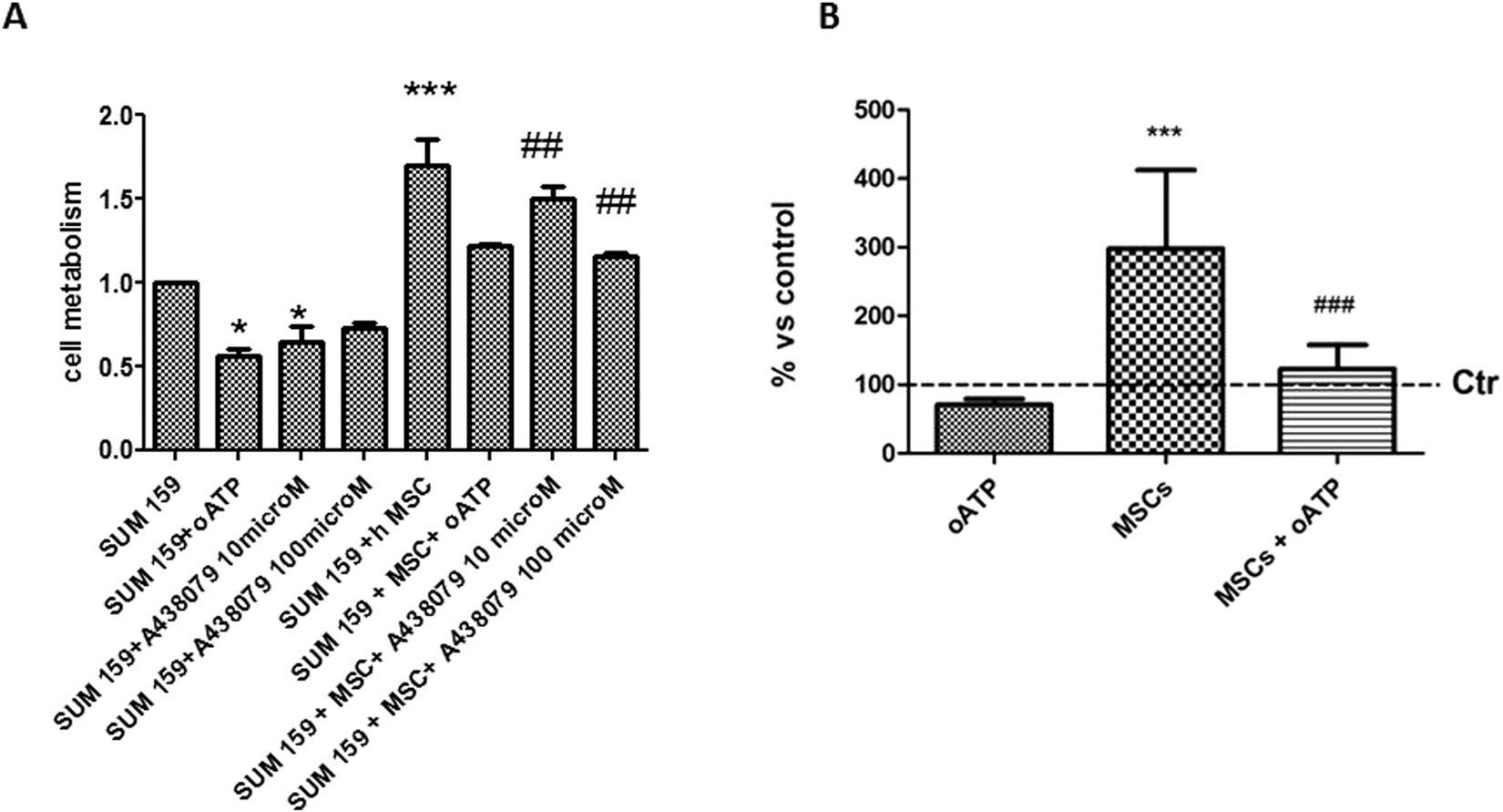
Effect of 100 µM oATP on SUM159PT cells. MSC coculture induced a significant increase in tumor cell metabolism (**A**) and growth (**B**). oATP, when given to the coculture, completely reverted the metabolism and the cellular growth to control values (**A**,**B**). When administered alone, oATP was able to induce an impairment in metabolism rate (A), but not to affect SUM159PT growth (B, percentage of increase with respect to control, Ctrl). The specific inhibitor of P2X7, A438079 induced a dose dependent inhibition on cell metabolism given to SUM159PT either alone or in coculture with MSC (A). One-way ANOVA with Bonferroni as post-hoc test: *p < 0.05 vs SUM159PT; ***p < 0.001 vs SUM159PT; ^##^p < 0.01 vs SUM159PT + MSC; ^###^p < 0.001 vs SUM159PT + MSC.

In light of these data, we addressed whether a tonic inhibition of P2X signaling, and P2X7 in particular, would modulate the metabolic activity of cancer cells. A general P2X receptor inhibitor (100 µM oATP) was administered to SUM159PT cells, either cultured alone or in the presence of MSCs for 48 hours, and metabolic activity was observed. SUM159PT co-cultured with MSCs showed a significant increase in cellular metabolism, in line with previously observed increase in cell number. Exposure of cancer cells (either alone or in co-culture with MSCs) to oATP significantly reduced cancer cell metabolism (Fig. 2A). When cells were administered a P2X7 specific inhibitor, A438079, metabolic activity was reduced in a dose dependent manner, thus suggesting a major role played by P2X7 on the modulation of cancer cell metabolism, following exposure to ATP-enriched TME milieu. Similar results were obtained when MSC cultured alone were exposed to 100 µM oATP (cell metabolism: MSC with oATP 0.5958 ± 0.1578,values normalized to untreated MSC cells).

We then focused our attention on the modulation of proliferation by MSC-derived ATP through P2X signaling. oATP completely reverted cancer cell proliferation increase induced by MSC co-culture (Fig. 2B), thus confirming, also in a breast cancer model, evidence in literature suggesting a role played by tonically activated P2X (and P2X7 in particular) in favoring cell growth^20,21^.

### P2X signalling inhibition reduces MSC-mediated increase in breast cancer cancerogenic potential *in vitro*

**Figure 3:**
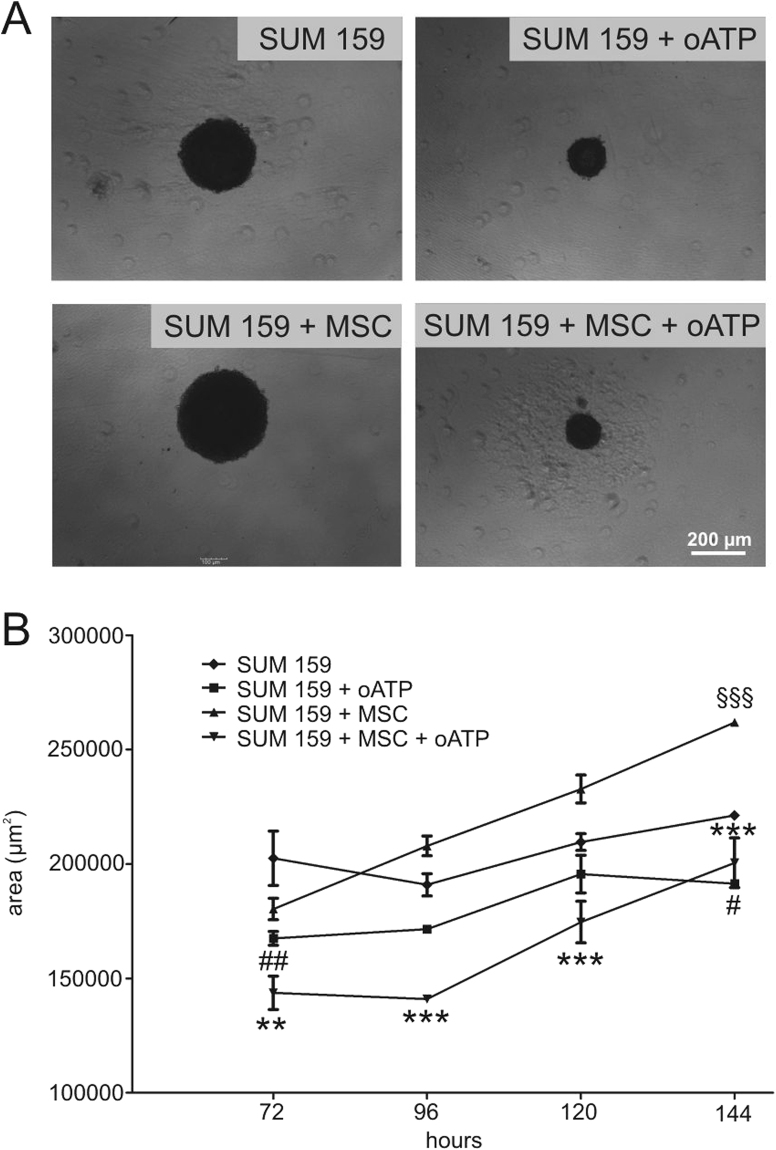
After SUM159PT culture, alone or with MSC, the effect of 100 µM oATP was evaluated on sphere formation capability. As showed in the representative photo (**A**) and quantified by measuring sphere area (**B**), MSC significantly increased the capability of SUM159PT to form mammosphere. oATP markedly inhibited mammosphere formation and growth either when given SUM159PT alone or to cocultures. Two-way ANOVA with Bonferroni as post-hoc test: **p < 0.01 vs SUM159PT + MSC; ***p < 0.001 vs SUM159PT + MSC; ^##^p < 0.01 vs SUM159PT; ^#^p < 0.05 vs SUM159PT; ^§§§^p < 0.001 vs SUM159PT.

In order to be further closer to the pathophysiological condition, a 3D system was set up and the formation of mammosphere evaluated. Exposure of cancer cells to MSCs induced an increase in spheroid size, measured by area size quantification, indicating a trophic effect in cancerogenic potential. The administration of oATP completely abolished the effect of MSCs and markedly inhibited the breast cancer cells’ capability of mammosphere formation (Fig. 3).

### P2X signalling inhibition reduces breast cancer invasiveness potential *in vitro*

**Figure 4:**
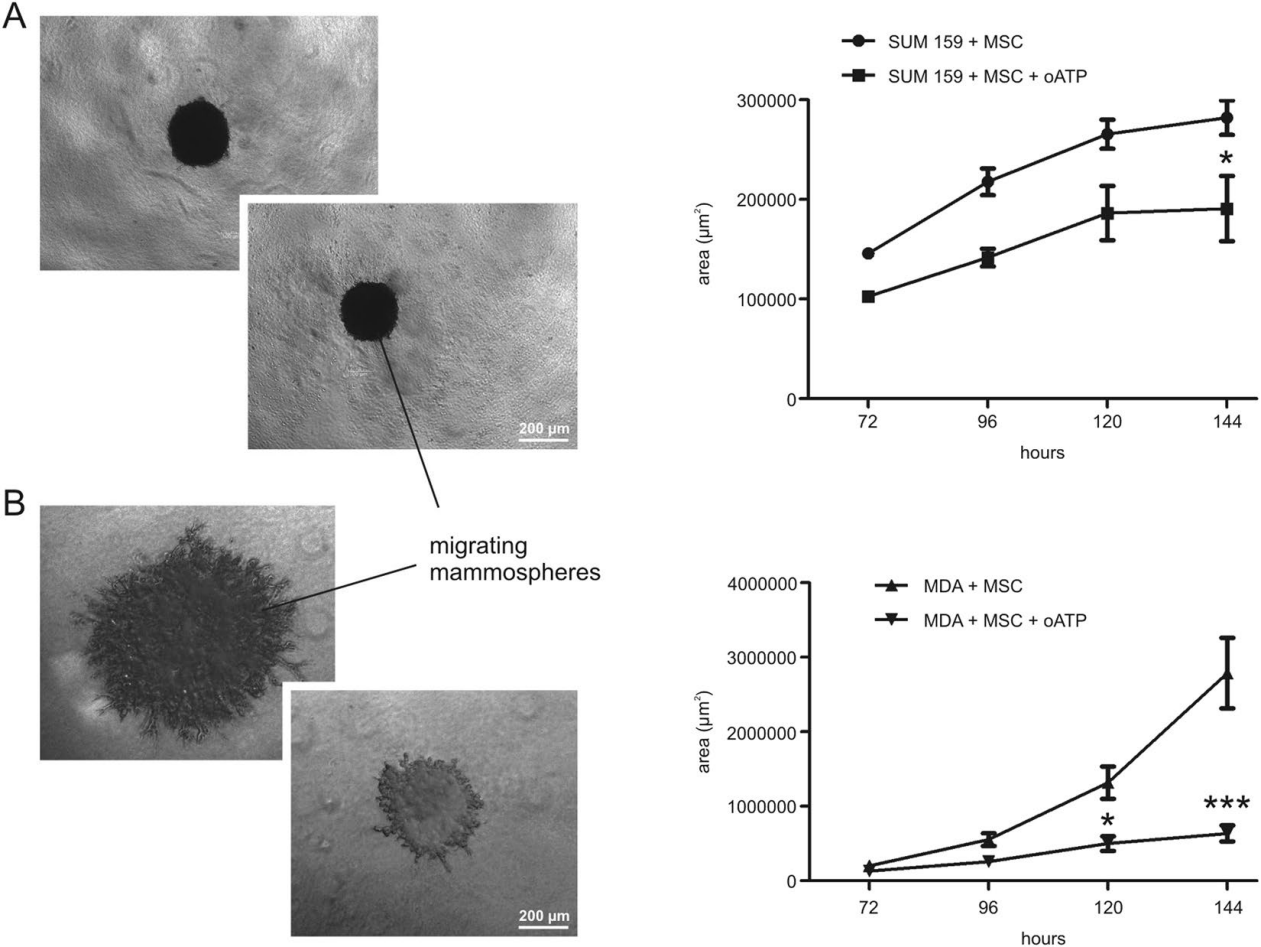
The effect of 100 µM oATP on invasive potential of SUM159PT and MDA-MB-231 cocultured with MSC. The invasiveness potential was quantified by measuring the area occupied by migrating mammospheres. SUM159PT, as indicated by the representative photo and the graph, showed a very low invasion, inhibited by the administration of oATP (**A**). On the other side MDA-MB-231 showed a very marked cellular migration from mammospheres, completely abolished by oATP treatment. Two-way ANOVA with Bonferroni as post-hoc test: *p < 0.05 vs SUM159PT + MSC (**A**) or MDA + MSC (**B**); ***p < 0.001 vs MDA + MSC.

The effect of oATP on metastatic potential was investigated using a 3D model of sphere invasion with two different human cell types characterized by different invasiveness potential (SUM159PT and more invasive MDA-MB-231), either in the presence/absence of MSCs. As expected, MDA-MB-231 showed much more marked invasiveness characteristics than SUM159PT, as indicated by representative photographs of invading mammospheres and their quantification (Fig. 4A).

oATP significantly inhibited the SUM159PT invasion after 144 hours of 3D culture. When an invasive cell line was used (MDA-MB-231), the effect of oATP was still present and even more marked, decreasing the metastatic potential of breast cancer cells after 120 hours of 3D culture.

The photographs in Fig. 4 clearly show the difference in invasiveness between SUM159PT and MDA-MB-231. In particular, migrating cells are indicated by branching from the core of the spheroid in presence of MSCs and are evident in MDA-MB-231 model and to a much lesser extent in SUM159PT.

## Discussion

In this study, we demonstrated that the trophic effect of MSCs on breast cancer growth is at least exerted via ionotropic purinergic signaling modulation. Our data sheds light on previously hypothesized role for organelle shedding in the MSC-mediated effects on cancer growth^22^. In particular, our data suggest a potential role played by MVs in the modulation of purinergic signaling between MSCs and cancer cells.

Whether this is due to soluble factors released by MVs or to MV-mediated receptor intercellular translocation, requires further investigation. In particular, data from the intracellular calcium response analysis seems to suggest that upon coculture, a significantly higher percentage of P2X expressing cells is observed. Whether this is due to receptor translocation or upregulation of selected P2X receptors expression (and in particular which P2X receptors) still needs to be eludicated. The inhibition of this system induced a significant reduction in tumor growth and metastatic potential, indicating the purinergic signaling system as a potential target for therapeutic intervention. It is known that MVs and exosomes contain high levels of ATP, which may be released in the tumor microenvironment and lead to purinoceptor activation^23^.

It is well known that MSCs are recruited to the site of tumorigenesis by many chemotactic factors secreted from cancer cells such as matrix metalloproteinases (MMPs), growth factors, and inflammatory cytokines. MSCs have a role on growth and progression of different tumors including breast cancer^24^. The mechanism of MSCs stimulation has been investigated and many hypotheses have been formulated but no single mechanism has yet been elucidated.

Malignant tumors such as breast cancer are characterized by a strong inflammatory response, in which ATP accumulates within the TME. While it is generally acknowledged that treatment with high levels of ATP or ATP analogues has a strong cytotoxic effect on some neoplasia^25^, it is also clear that low ATP doses (as occurring, for example, during spontaneous release of this nucleotide from virtually every cell type) have a growth-promoting effect. Depending on the P2 receptor subtypes expressed, tumor cells may be more sensitive to the death-inducing rather than the trophic effect of ATP.

We therefore investigated the role played by P2 purinergic receptors on cancer cells, studying the intracellular calcium dynamics when cultured either in the presence or absence of MSCs. In particular, given changes in the cytosolic concentrations of calcium can induce signalling pathways that regulate a broad range of cellular events, including those important in tumorigenesis, we observed both the basal levels of intracellular calcium in cancer cells as well as their acute response following exogenous ATP challenge. Basal intracellular calcium levels of SUM159PT were not influenced when cultured in microfluidic connection with MSCs. Moreover, quantitative analysis of the intracellular calcium response following exogenous ATP stimulation suggests a strong role of P2X but not P2Y in modulation of cancer cell response.

Many studies demonstrated a correlation between P2X receptor activation by ATP and tumor growth inhibition in different cancer types, such as leukemia^26^, melanoma^27^, squamous cell skin cancer^28^, lung cancer^29^ and cervical cancer^30^. The P2X7 receptor is most widely accepted as the purinergic receptor mediator of apoptotic or necrotic cell death, as initially suggested by early experiments in mouse tumor cell lines, where ATP was shown to trigger cell death via necrosis or apoptosis, depending on the cell type. Nevertheless, there’s a growing amount of literature indicating that tonic, as opposed to pharmacological, stimulation may have a trophic, growth-promoting, rather than cytotoxic effect^14,15^. According to literature, the tumor microenvironment is rich in ATP^12,13,19^. Data on the role of purinergic signaling in cancer, in response to extracellular levels of ATP are not conclusive, likely due to the presence of many different purinoceptors and tumors^31^. In particular, in breast cancer, a micromolar concentration range of ATP induces a favorable effect in migration and invasion^31^. In line with existing literature^32^, our data indicate that the levels of ATP reached in the presence of MSCs are in the micromolar range, in fact we found stable basal levels of Ca^2+^ even in the presence of MSCs (Fig. 1E). This finding is further supported by the results observed following administration of 1 mM exogenous ATP, inducing an increase in the number of responsive cells.

The inhibition of tumor growth was significant when cells were challenged by a general P2X receptor inhibitor (oATP); similar effects were obtained using a more specific P2X7 inhibitor (A438079). This effect is particularly prominent when evaluating MSCs action on cancer cells, indicating that P2X7 may be an important player in the regulation of tumor growth, as demonstrated by recent published evidence^20,33^, but, at least in breast cancer *in vitro*, other purinergic receptors concur to this process. The contribution of the single P2X receptors should be therefore further investigated.

oATP was able to completely abolish the metastatic potential of SUM159PT, but most importantly inhibited the metastatic potential of MDA-MB-231 cells, a very invasive cell line, in presence of MSCs. These results, if confirmed *in vivo*, are potentially very promising for clinical implications in the treatment of breast cancer.

In conclusion, all these data, taken together clearly indicate a role of purinergic ionotropic receptors in breast cancer. In order to evaluate potential therapeutical approaches, data obtained clearly shows the need to address the role played by MSCs in the cancer model, which so importantly positively modulate the oncogenic potential of breast cancer cells.

The inhibition of purinergic receptors should be further investigated in *in vivo* models using xenograft tumor transplants in presence of human MSCs. If our data will be confirmed, promising clinical applications can be evaluated.

## Methods

### Human breast cancer cells

SUM159PT cells (human breast cancer cell line) were purchased from Asterand (Royston, UK). Cells were maintained in Ham’s F12 Nutrient Mix (Thermo Fischer Sc., MA, USA) supplemented with 5% FBS (Thermo Fischer Sc., MA, USA), hydrocortisone 1 μg/mL (Sigma-Aldrich, Milan, Italy), Insulin 5 μg/mL (Sigma Aldrich, Milan, Italy), HEPES 10 mM (Thermo Fischer Sc., MA, USA), and PenStrep. Cells were sub-cultured according to the manufacturer’s instructions.

MDA-MB-231 (human breast adenocarcinoma cell line), a kind gift of ICLC-Biologic Bank and Cell Factory, (Genova, Italy) were maintained in DMEM high glucose (Sigma-Aldrich, Milan, Italy) supplemented with 10% FBS (Thermo Fischer Sc. 10270), L-glutamine 2 mM, 1X MEM Non-Essential Amino Acids Solution (Thermo Fischer Sc. 11140035), HEPES 10 mM and PenStrep.

### Human mesenchymal stem cells

Primary human adipose-derived MSCs were purchased from Cellular Engineering Technologies. Cells were phenotyped via Fluorescence-activated cell sorting (CD14−, CD45−, CD29+, CD44+, CD90+, CD105+). Cells were cultured in DMEM low glucose (Thermo Fischer Sc., MA, USA) supplemented with 10% FBS (Thermo Fischer Sc., MA, USA), HEPES 10 mM and PenStrep.

### Co-cultures

Cancer cells were cultured alone or co-cultured with MSCs, and treated with oxiATP (Sigma-Aldrich, Milan, Italy). Co-cultures of MSCs and cancer cells were made on microfluidic chips as previously reported^34^. After overnight attachment of the cells, the medium was replaced with fresh cancer cell medium, allowing the communication between the chambers. Treatment with oxiATP (oATP) was administered 48 and 24 h before each experiment/assay.

### Quantitative evaluation of intracellular calcium dynamics

Cultures were loaded for 35–40 min at 37 °C with 2 μM Fura-2-AM in Krebs–Ringer solution buffered with HEPES, 125 mM NaCl, 5 mM KCl, 1.2 mM MgSO_4_, 2 mM CaCl_2_, 10 mM glucose, and 25 mM HEPES (pH 7.4), and were washed twice with pre-warmed Krebs–Ringer solution before recordings were made. The recording setup comprised an inverted microscope (Axiovert 100, Zeiss, Germany) equipped with a Ca^2+^ imaging unit. A Polychrome IV (TILL Photonics, Germany) was used as a light source. Fura-2 fluorescence images were collected using a PCO Super VGA SensiCam (Axon Instruments, CA, USA) and analyzed with TILL Vision Software (TILL Photonics, Germany). Single-cell 340/380 nm fluorescence ratios, acquired at a sampling frequency of 1–4 s^−1^, were analyzed with Origin 6.0 (Microcal Software Inc., MA, USA).

### Open microfluidic network chip fabrication

The microfluidic chips were made by casting poly(dimethylsiloxane) (PDMS) (Sylgard® 184, Dow Corning, Midland, MI) onto micro-structured molds. The molds were fabricated by etching 150 µm deep structures into 4 inch Si wafers (Siltronix, Geneva, Switzerland) using standard UV photolithography and deep reactive ion etching (AMS-200SE, Alcatel Micro Machining Systems). Vapor deposition of perfluorosilane (1 H,1 H,2 H,2H-perfluorooctyltrichlorosilane, ABCR, Karlsruhe, Germany) onto the Si mold generated a non-adhesive layer for the PDMS casting. The mixed PDMS components were dispensed onto the Si mold and cured over night at 60 °C. The cured PDMS was then peeled off the Si mold and the individual microfluidic chips were separated using a sharp knife.

To prepare the microfluidic chips for the seeding of cells, the surface of the chips carrying the microstructures was treated with an oxygen plasma for 30 s (Technics Plasma 100-E, Florence, KY) at 200-W coil power. This treatment oxidized the surface of the PDMS and a subsequent coating with polylysine was performed by dispensing a 0.5 mg/mL solution of polylysine onto the surface and incubation overnight at room temperature. Washing with PBS and water was performed after the polylysine incubation and the chips were then dried under a stream of nitrogen. Cell suspensions were then added onto the chambers and the microfluidic chips incubated in Petri dishes together with a few mL of water next to them to prevent evaporation of liquid from the cell chambers.

### Metabolic activity

Cell metabolism was measured by Thiazolyl Blue Tetrazolium Blue (MTT) assay (Sigma-Aldrich, Milan, Italy). The reagent was administered 0.5 mg/mL in P35/P60 dishes. After 3 h 30 min, the medium was removed, and cells containing the insoluble formazan were solubilized with 100% DMSO. Absorbance at 550 nm was then measured using a Tecan Infinite F500 reader.

### Vitality analysis

SUM159PT or MDA-MB-231 cells were cultured alone or in co-culture with MSCs in two different ways, in order to address the difference between microvesicle permissive or non-permissive systems (Fig. 1A,C). In the non-permissive system, MSCs (10,000 cells/cm^2^) were plated in a transwell insert with 0.4 µm pore size (Corning, NY, US) and SUM159PT (10,000 cells/cm^2^) in the chamber below. After 100 μM oATP treatment, administered to either cancer cells cultured independently or co-cultured with MSCs, cancer cells were detached and the number of viable cells were assessed using trypan blue solution 0.4% (Sigma Aldrich, Milan, Italy).

### Migration

Motility of cancer cells co-cultured with MSCs and treated with oATP was measured using the Neuro Probe MBB96 chemotaxis chamber. Cancer cells were harvested, counted, and seeded in the top plate at 50.000 cells/well and 200 µL/well. In the lower plate, 420 µL medium containing EGF at 20 ng/mL was dispensed into the wells. Migrating cells remained trapped in the framed polycarbonate filter (8 μm pores) preventively coated with poly-L-lysine hydrobromide (Sigma-Aldrich, Milan, Italy). After the over-night migration, the framed filter was washed with 1x PBS and trapped cells were stained with calcein 2 µM for 30′ at 37 °C. Fluorescence was then measured using a Tecan Infinite F500 reader (λ_ex_ = 480 nm, λ_em_ = 510 nm).

### 3D mammosphere formation

After co-culture of MSCs with cancer cells and treatment with oATP as described above, cancer cells where detached and plated for the mammosphere formation assay. Formation of mammospheres was performed using a Cultrex 3D Spheroid Fluorometric Proliferation assay (Trevigen, MD, USA). According to the manufacturer’s instructions, 2500 cells/well were plated. Growth of mammospheres was observed after 72 h of incubation at 37 °C, and measured every 24 h until 144 h. The area of spheres was measured using the Motic Images Advanced 3.2 software.

### 3D mammosphere Invasion

After co-culture of MSCs with cancer cells and treatment with oATP as described above, cancer cells where detached and plated for the mammosphere invasion assay. Invasion of mammospheres was performed using a Cultrex 3D Spheroid BME Cell Invasion assay (Trevigen, MD, USA). According to the manufacturer’s instructions, 2500 cells/well were plated. Invasion of mammospheres was observed after 72 h of incubation at 37 °C, using 160 ng/mL EGF as a chemoattractant, and measured every 24 h until 144 h. The area of spheres was measured using the Motic Images Advanced 3.2 software.

### Statistical analysis

Data were analyzed using one- or two-way ANOVA as appropriate. Bonferroni was used as post-hoc test.
